# Diverse and abundant resistome in terrestrial and aquatic vertebrates revealed by transcriptional analysis

**DOI:** 10.1038/s41598-020-75904-x

**Published:** 2020-11-02

**Authors:** Yan-Mei Chen, Edward C. Holmes, Xiao Chen, Jun-Hua Tian, Xian-Dan Lin, Xin-Cheng Qin, Wen-Hua Gao, Jing Liu, Zhong-Dao Wu, Yong-Zhen Zhang

**Affiliations:** 1 Zhongshan School of Medicine, Sun Yat-Sen University, Guangzhou, 510080 China; 2 Shanghai Public Health Clinical Center, Fudan University, Shanghai, China; 3 Department of Zoonosis, Chinese Center for Disease Control and Prevention, National Institute for Communicable Disease Control and Prevention, Changping Beijing, China; 4 School of Life and Environmental Sciences and School of Medical Sciences, Marie Bashir Institute for Infectious Diseases and Biosecurity, The University of Sydney, Sydney, Australia; 5 College of Marine Sciences, South China Agricultural University, Guangzhou, Guangdong China; 6 Wuhan Center for Disease Control and Prevention, Wuhan, Hubei China; 7 Wenzhou Center for Disease Control and Prevention, Wenzhou, Zhejiang China

**Keywords:** Antimicrobial resistance, Microbial ecology, Microbiology, Ecology

## Abstract

Despite increasing evidence that antibiotic resistant pathogens are shared among humans and animals, the diversity, abundance and patterns of spread of antibiotic resistance genes (ARGs) in wildlife remains unclear. We identified 194 ARGs associated with phenotypic resistance to 13 types of antibiotic in meta-transcriptomic data generated from a broad range of lower vertebrates residing in both terrestrial and aquatic habitats. These ARGs, confirmed by PCR, included those that shared high sequence similarity to clinical isolates of public health concern. Notably, the lower vertebrate resistome varied by ecological niche of the host sampled. The resistomes in marine fish shared high similarity and were characterized by very high abundance, distinct from that observed in other habitats. An assessment of ARG mobility found that ARGs in marine fish were frequently co-localized with mobile elements, indicating that they were likely spread by horizontal gene transfer. Together, these data reveal the remarkable diversity and transcriptional levels of ARGs in lower vertebrates, and suggest that these wildlife species might play an important role in the global spread of ARGs.

## Introduction

Antibiotics have made an enormous contribution to public health, protecting populations against bacterial pathogens and extending life expectancy. However, concomitant with the development of antibiotics has been the rise of antibiotic resistance^1^. The misuse of antibiotics in humans and animals has massively accelerated the evolution of resistance against a diverse range of antibiotics^2^. Antibiotic-resistant pathogens have emerged frequently in humans and domestic animals, presenting a substantial threat to public health, that will remain for the foreseeable future^3–7^.

Antibiotic resistance, including that to major human pathogens, is abundant in the environment and has resided there for an extended period of time^1,8–11^. Antibiotic-resistant bacteria have been observed in both wild animals and their natural habitats, including those with little direct human interaction^12–16^. Because wildlife are ubiquitous, and some are able to move long distances (e.g., birds, fish), it is possible that they play an important role in the global spread of antibiotic-resistant bacteria and resistance genes^17,18^. As a case in point, aquatic birds have been implicated in the worldwide dissemination of beta-lactam and tetracycline antibiotic resistance genes (ARGs), likely through contact with contaminated water^19–21^. To date, ARGs has been identified in the microbiota of multiple wildlife species^22^. Interestingly, the diversity and abundance of the antibiotic resistome in wildlife varies according to the animal species sampled and their habitats. For example, wastewater/sludge from wastewater treatment plants harbored more abundant ARGs than water, sediment and soil from natural environments^23^. Similarly, birds feeding at wastewater treatment plants harbor a greater diversity and abundance of ARGs than those feeding at more pristine environments^24^. Nevertheless, only a few studies of the diversity, abundance and dissemination of antibiotic resistome in wildlife have been performed to date, largely focusing on mammals and birds^8,24–27^. While lower vertebrates such as fish, amphibians and reptiles are more speciose than birds and mammals, far less is known about their resistome.

The misuse of antibiotics, leading to the selection of antibiotic-resistant bacteria, is one of the key drivers of global ARG prevalence^28,29^. The antibiotic resistome is also able to expand rapidly even in the absence of continuous selection pressure due to horizontal gene transfer (HGT) among bacterial species^30^, especially for ARGs encoded in mobile genetic elements (MGEs)^31,32^. Importantly, ARGs might be transferred between human pathogens and antibiotic-resistant bacteria not known to infect humans via HGT in wildlife and their habitats^21,22^. Due to the increasing interactions among humans, domestic animals and wildlife, the exchange of ARGs among species is likely to be commonplace^17,22^. Hence, wildlife may play an important role in the evolution and spread of antibiotic resistome.

Only small-scale studies of ARGs in wildlife have been conducted to date so that the picture of ARG diversity and abundance in these species is unclear. To better understand the diversity and evolution of ARGs, DNA-based metagenomics has been increasingly used as an analytical tool^1,33–35^. Despite its usefulness, this technique is unable to distinguish recently deactivated resistance genes from those that are still functional. This is important since resistance genes, especially those recently acquired, are likely to impose a metabolic burden fitness cost in the absence of antibiotics and hence are expected to be selectively eliminated^36,37^. In comparison, RNA-based meta-transcriptomics (i.e. RNA shotgun sequencing) can assess the entire set of functionally active genes (i.e. transcribed) without bias^38^. To date, however, meta-transcriptomics has only been used in a few studies of antibiotic resistance^39–41^, particularly wildlife^24^.

We collected meta-transcriptomic data from a broad range of wild lower vertebrates residing in a range of habitats from ocean to land. We used these data to investigate the diversity and abundance of antibiotic resistome transcribed in the whole microbiome in these wild animals, and to identify the potential mechanisms of ARG distribution and transmission among wildlife.

## Results

### The antibiotic resistome in lower vertebrates

**Figure 1:**
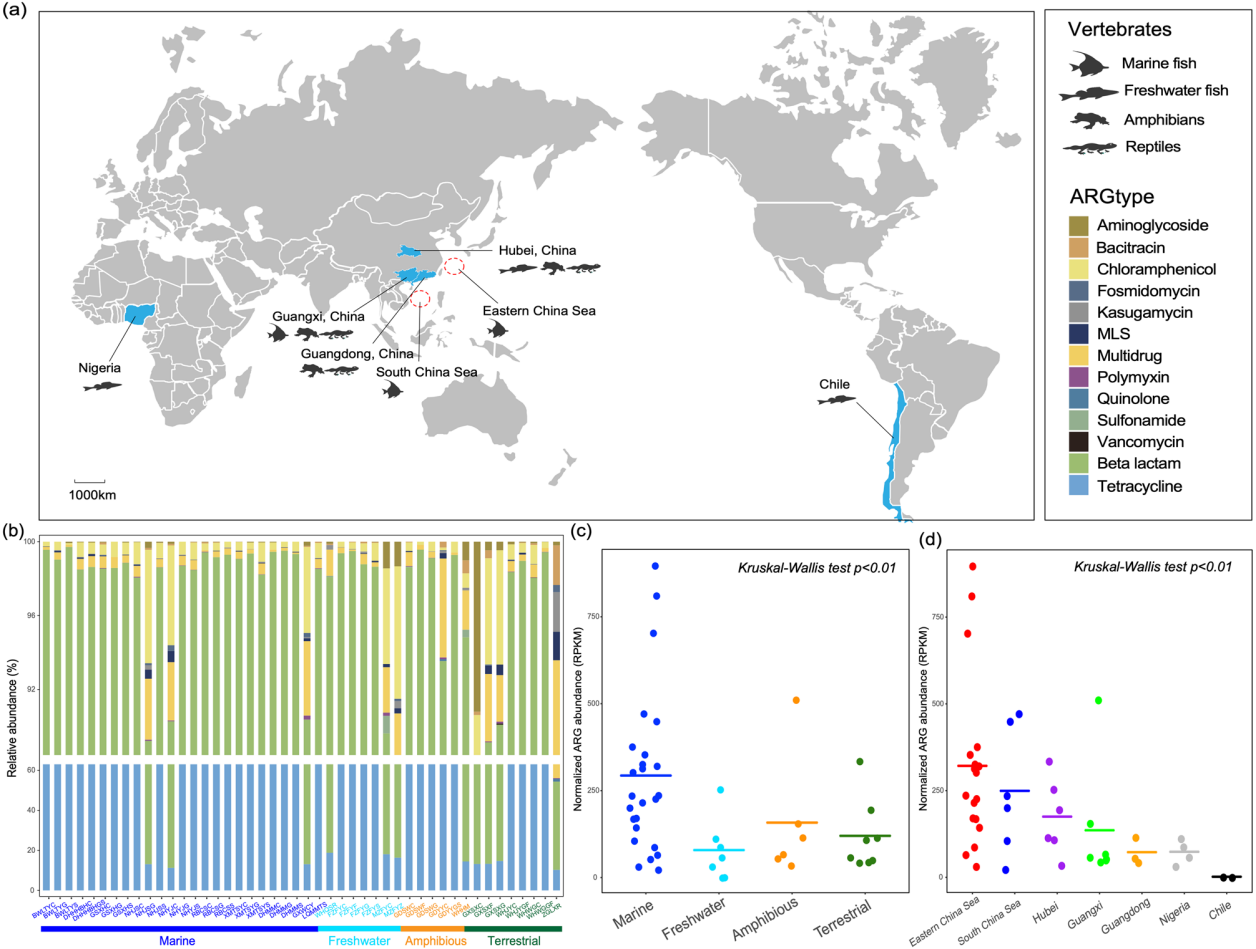
Antibiotic resistome in vertebrate meta-transcriptomic libraries. (**a**) Sample locations and sample types of the meta-transcriptomic data obtained here. (**b**) Relative abundance of ARG types in each library. Colors for each ARG type are presented in the top right panel of the figure. MLS, macrolide, lincosamide and streptogramin. (**c**–**d**) Total ARG abundance (RPKM) per library, stratified by habitat (**c**) and sampling site (**d**). Cross bars indicate the mean values. Differences between groups were estimated using a Kruskal–Wallis test.

A total of 46 meta-transcriptomic libraries were chosen from our previous study of RNA viromes in lower vertebrates^42^. Animals from which these libraries were produced were collected from mainland and marine environments in China, as well as from Chile and Nigeria (Fig. 1a). In total, these data comprised vertebrates sampled from terrestrial (five species), amphibious (three species), freshwater (more than three species) and ocean environments (14 species), covering seven vertebrate classes in the phylum Chordata (Fig. 1a; Table S1). RNA libraries were generated from different tissue types (i.e., gut, liver, gill, lung, and mixed tissues; Table S1). An average number of 4 million adaptor- and quality-filtered reads (range 2–5 million) were obtained for each library. Of these, an average of 0.02% (range 0.001–0.07%) of reads were mapped to ARGs using Diamond Blastx^43^.

A total of 194 ARGs associated with phenotypic resistance to 13 types of antibiotic were identified in these animals (Table S2). Of all these ARG types, genes encoding resistance toward tetracycline and beta-lactam antibiotics were the most abundant (i.e. transcription level) across all libraries, while those conferring resistance to chloramphenicol, aminoglycoside, macrolide-lincosamide-streptogramin (MLS), fosmidomycin and multidrug were also found in all classes of vertebrates despite their relatively low abundance (Fig. 1b, Table S2). Lizards inhabiting terrestrial environments (libraries GXSXC, GXSXF, GXSXG, and ZGLXR) had the most diverse composition of resistance genes, harboring a relatively high proportion of genes providing resistance to aminoglycoside, macrolide-lincosamide-streptogramin (MLS), as well as conferring multidrug resistance. In contrast, fish samples, especially those from marine fish, generally had similar resistome compositions, with the exception of three samples from cartilaginous fish and lancelets (Fig. 1b). Twenty ARGs contributed almost 90% of total abundance across all libraries (Fig. S2), particularly in marine fish.

To better understand the association between the resistome and ecological niche, meta-transcriptomic libraries were separated into four groups (“habitats”) according to host habitat: (1) terrestrial, (2) amphibious, (3) marine and (4) freshwater (Table S1). Remarkably, total ARG abundance varied across sampling sites and host habitats (Kruskal–Wallis test *p* < 0.01; Fig. 1c,d). In particular, marine fish had the highest ARG abundance (average 293.4 reads per kilo bases per million reads (RPKM)), whereas the lowest ARG level was observed in freshwater fish (average 79.1 RPKM) (Fig. 1c). Consistent with this pattern was that high ARG levels were observed in the Eastern China Sea (average 321.4 RPKM) and South China Sea (average 249.3 RPKM), but much lower in Nigeria and Chile (average 1.78 RPKM) where the freshwater fish were sampled (Fig. 1d).

As the samples were collected from different animal tissues, we also investigated the association between ARG and tissue type, comparing libraries among habitats and sites in individual tissues. This revealed no significant difference in ARG abundance between tissue types (Kruskal–Wallis test *p* = 0.19). Additionally, although the small number of libraries per tissue type per habitat/site precluded a statistical analysis, graphically the results suggested comparably high ARG levels in different tissues in marine fish (Fig. S3). Given that the sampling sites also largely represented the host habitat (e.g. all samples in Eastern China Sea and South China were from marine environments, while those in Nigeria and Chile were all freshwater fish), our statistical analysis focused on the association between ARG and habitat.

### Effect of ecological niche on ARG diversity and clustering

**Figure 2:**
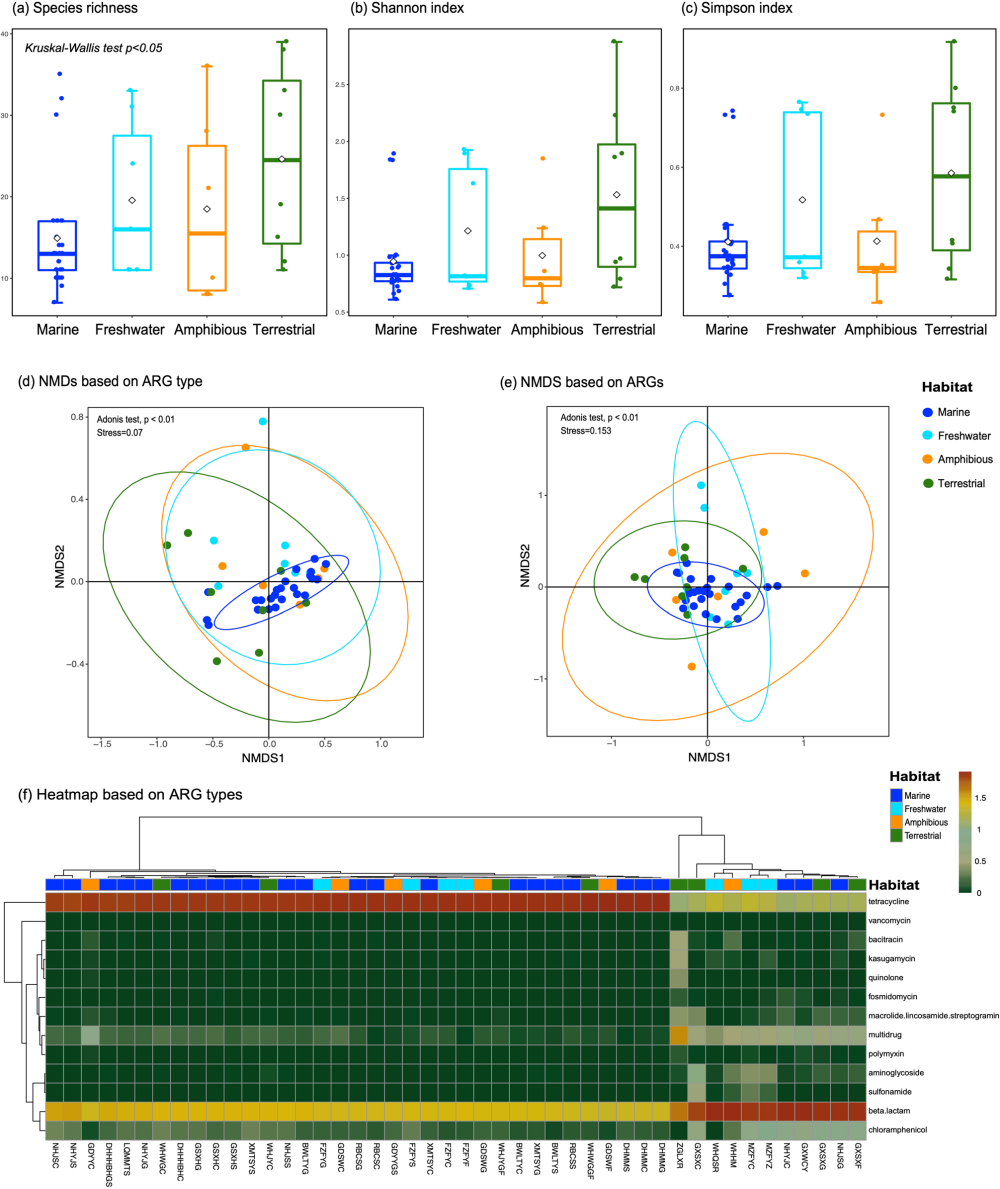
ARG diversity and clustering in vertebrate samples across habitats. (**a**) Species richness. (**b**) Shannon diversity index. (**c**) Simpson diversity index. The horizontal box lines represent the first quartile, the median, and the third quartile. Whiskers denote the range of points within the first quartile − 1.5 × the interquartile range and the third quartile + 1.5 × the interquartile range. Diamond represents the mean values. Differences between groups were estimated using a Kruskal–Wallis test. (**d**–**e**) Non-metric multidimensional scaling (NMDS) analysis on ARG type (**d**) and gene (**e**) levels. Ellipses were drawn at a confidence level of 0.95. Effect of habitat on resistome clustering was assessed using an Adonis test. (**f**) Heat map on ARG type level. Bray–Curtis dissimilarity and Pearson correlation coefficients were used to hierarchically cluster (using the UPGMA method) samples and ARGs, respectively.

We further assessed the diversity and clustering patterns of ARGs in these animals across habitats. Species richness and two alpha diversity indices (Shannon and Simpson) were therefore calculated for the gene level resistomes in each sample (Fig. 2a–c). Both the mean and median richness and diversity indices were higher in terrestrial vertebrates than in other habitats, although a significant difference was only observed for species richness (Kruskal–Wallis test *p* < 0.05). It is likely that the terrestrial vertebrates not only possess greater resistome diversity but also a more homogeneous distribution of different ARGs. In contrast, marine fish had the lowest species richness and diversity of ARGs among the four habitats.

Importantly, the higher ARG diversity in terrestrial and amphibious vertebrates than that in marine fish was validated by the PCR analysis of individual specimens (Kruskal–Wallis test *p* < 0.001, Fig. S4). Of the 19 ARGs successfully amplified, we observed 164 and 76 occurrences in 27 amphibious and 17 terrestrial samples, respectively, while only 40 ARG occurrences were documented in 58 marine samples and 76 in 48 freshwater samples (Table S3). Similar results were observed in individual tissue types (Fig. S4).

There was a clear separation in resistome composition between samples from different ecological niches, with 12% of resistome variation explained at both the ARG type and gene levels (Adonis test, *p* < 0.01). Samples from marine fish clustered more closely together than those from other habitats (Fig. 2d,e), indicating that marine fish had more similar resistome compositions than samples from the other habitats. Resistome separation was mainly associated with the uniformly high proportion of tetracycline and beta-lactam resistance genes in marine fish (Fig. 2f). At both the ARG type and gene levels, the samples clearly separated into two groups, in which only three marine fish samples did not cluster with all other samples from the same habitat (Fig. 2f; Fig. S5). Remarkably, this clear separation of the marine fish resistome compared to those seen in other habitats was also observed at the level of individual tissues, although statistical analysis was precluded due to small sample size (Fig. S6). In sum, these data suggest that the antibiotic resistome in lower vertebrates is largely shaped by ecological niche, and that marine fish have a distinctive resistome compared to animals sampled from other habitats.

Notably, the SARG database grouped resistance genes into 23 ARG types (with clear resistance to specific antibiotics) and 1192 ARGs (specific resistance genes), including genes that require mutations to become drug resistant (Table S4). We included these genes in our analysis because we aimed to distinguish all potential resistance genes in these wildlife species. However, to take account of potential false-positive results, we repeated the analysis of resistome abundance, diversity and composition excluding these ARGs (that is, excluding *omp*36 and *omp*F). This did not impact the results in a meaningful manner (Fig S7).

### Dissemination of ARGs in wild vertebrates

**Figure 3:**
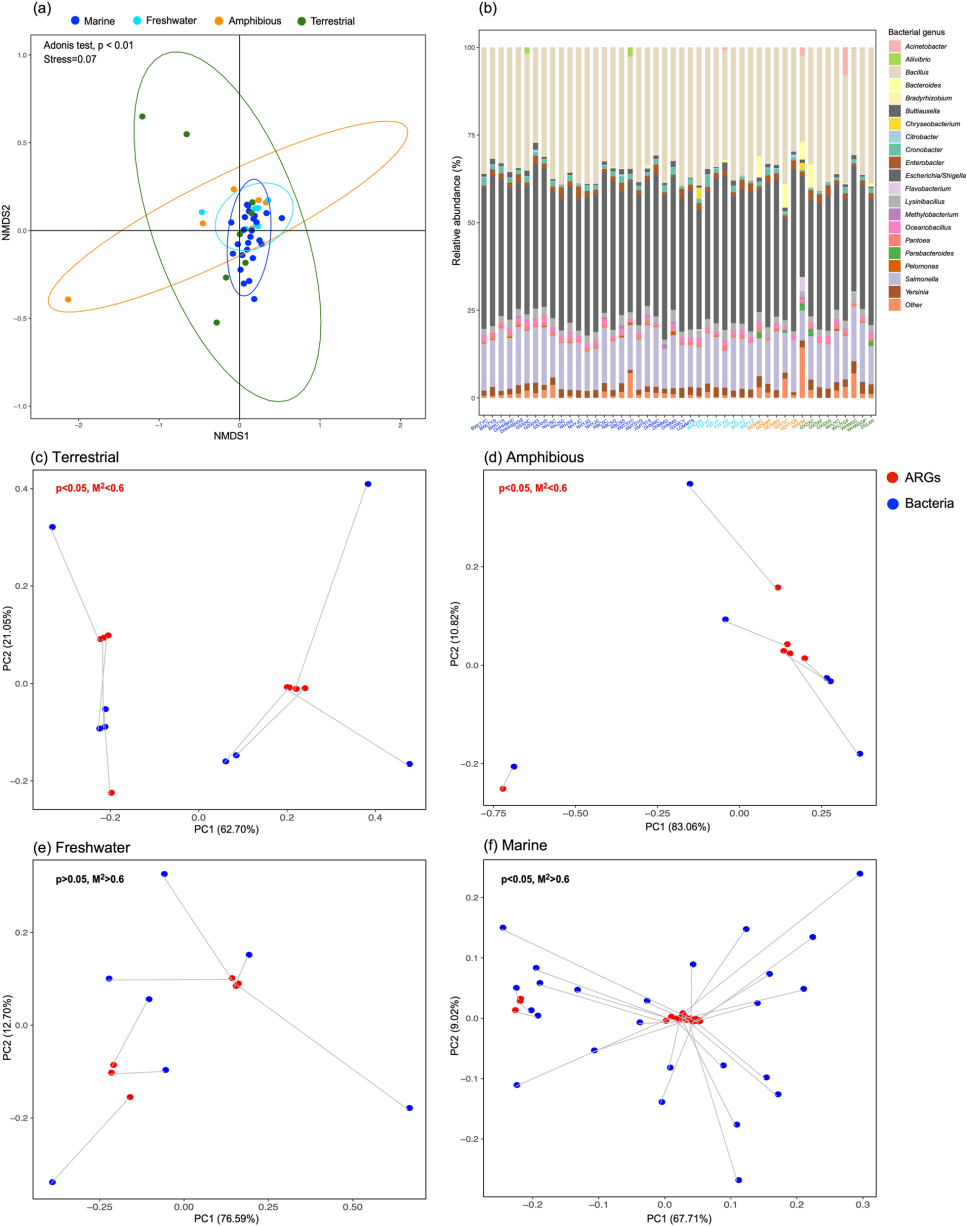
The active microbiome in wild vertebrates and their association with the resistome. (**a**) Non-metric multidimensional scaling (NMDS) analysis on bacterial genus level. Ellipses were drawn at a confidence level of 0.95. The effect of habitat on resistome clustering was assessed using an Adonis test. (**b**) Relative abundance of active bacterial genera in each library. (**c**–**d**) Procrustes analysis show the correlation between antibiotic resistome and the active bacterial communities in different habitats. Significant correlations were observed in terrestrial and amphibious vertebrates.

To better understand the circulation of ARGs in wildlife, we characterized the active microbial community in the species sampled (Table S5) using 16S rRNA transcripts, and assessed the effect of habitat on microbiome. In a similar manner to the antibiotic resistome, a more compact clustering of microbial community (Adonis test *p* < 0.01, 15.6%, Fig. 3a) with a similar community composition (Fig. 3b) was observed in marine fish compared to vertebrates in other habitats. *Bacillus*, *Escherichia* and *Salmonella* were the top three abundant bacterial genera in all libraries, especially in marine fish where they accounted for 82.3–91.3% of total bacterial abundance (Fig. 3b). The correlation between ARG metrics and bacterial communities were then determined in each habitat using both a Mantel test and Procrustes analysis. These revealed a looser correlation between the resistome and bacterial community in aquatic vertebrates (Mantel test, *p* > 0.05 and correlation coefficient *r* < 0.1; Procrustes analysis, *p* > 0.05 and/or *M*^2^ > 0.6), compared to terrestrial habitats (Mantel test, *p* < 0.05; Procrustes analysis, *p* < 0.05, *M*^2^ < 0.6; Fig. 3c–f; Table S6). Hence, wild vertebrates in different habitats differed not only in the composition of their microbiome and resistome, but also in the correlation between them.

**Figure 4:**
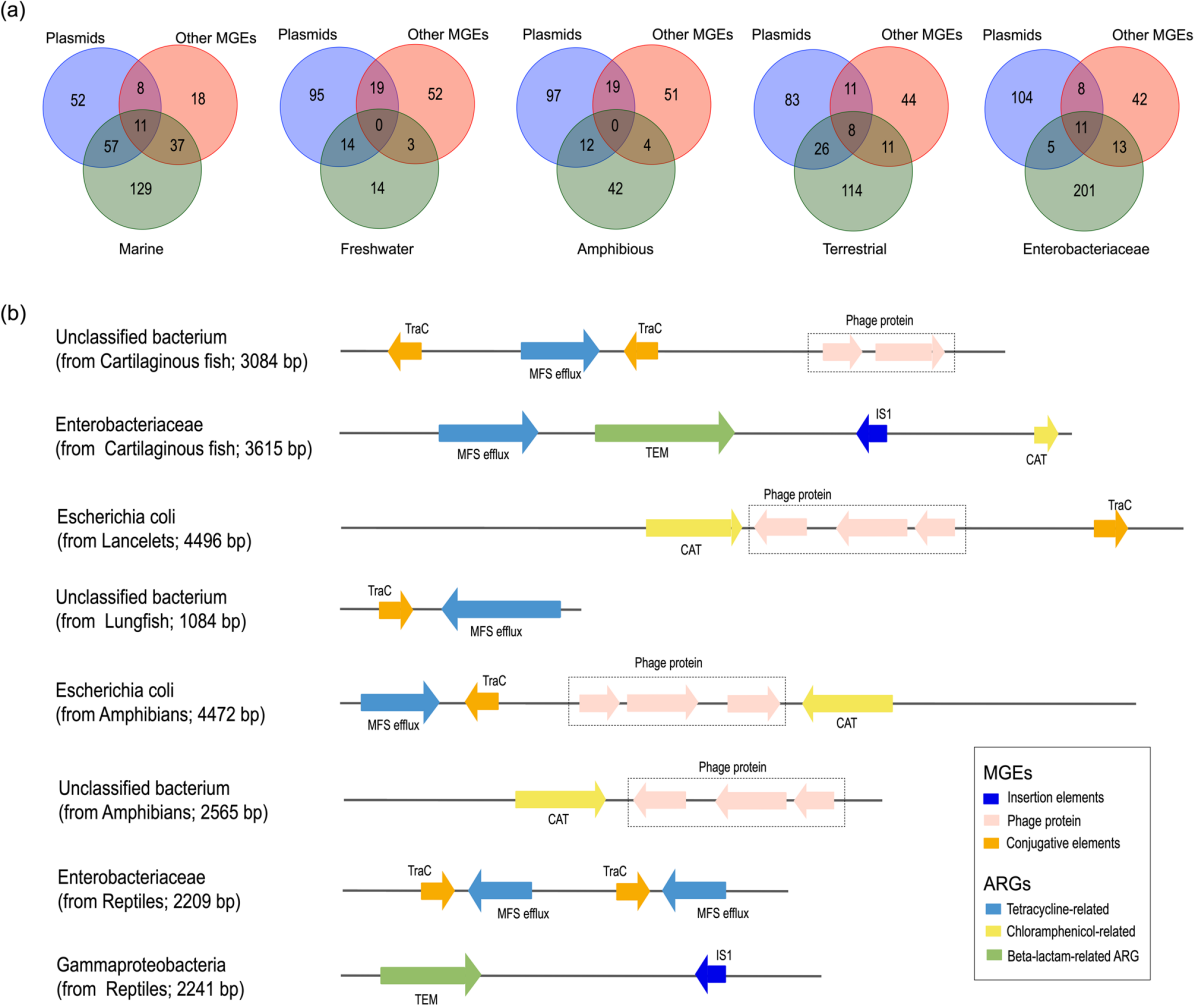
Potential mobility of ARGs in wild vertebrates. (**a**) Venn diagram showing the number of ARG contigs annotated to plasmids, other MGEs in each habitat and in contigs identified as *Enterobacteriaceae* fragments. (**b**) Representative alignments showing the co-localization of ARGs and putative MGEs in bacterial hosts.

To gain further insights into why wild animals from different habitats harbor different resistomes and how they disseminate resistance genes, we screened bacterial associated ARG contigs and looked for flanking MGEs indicative of HGT^41,44^. Accordingly, a total of 482 ARG contigs (> 80% amino acid identity with > 20% coverage) were annotated as bacterial fragments, most of which were carried by the phyla *Proteobacteria*, *Firmicutes* and *Bacteroidetes* (with the exception of those unclassified; Table S7). Among these, 38.0% of the ARG contigs were either very similar to plasmids or co-localized with other MGEs such as conjugative elements, insertion elements (i.e. transposable elements) and bacteriophage (Fig. 4a; Table S7). Tetracycline-, chloramphenicol- and beta-lactam-related ARGs, including those with > 99% amino acid identity to clinical isolates such as the *tet*A (YP_001687825), *bla*_TEM_ (GQ149347, DQ909059, AY040093) and *cat* (AF326777) genes, were those most frequently flanked by MGEs (Fig. 4b; Table S7). This is compatible with their possible transfer to or from other microbes. It also reduces the likelihood that they reflect reagent contamination.

Strikingly, fish harbored a greater percentage of mobile ARGs (54.8% for freshwater fish and 44.9% for marine fish) than amphibians (27.6%) and reptiles (28.3%) (Fig. 4a; Table S7). This result was consistent with the loose correlation between the microbiome and resistome in fish, and is suggestive of a higher frequency of HGT among aquatic populations. With respect to bacterial hosts, the mobile ARGs were mainly carried by bacteria from the *Enterobacteriaceae* (Fig. 4a), which may act as an important conduit for ARGs to move among wild animals.

## Discussion

A high diversity of ARGs have been identified in wildlife, particularly in those species that live in close proximity to humans, and hence that may be important in the evolution and dissemination of antibiotic resistance^45–47^. For instance, in the UK, a greater diversity and prevalence of resistance was found in *E. coli* isolated from rodents living in livestock farms than those from forests habitats^22^. Recently, 81 unique ARGs conferring resistance to nine classes of antibiotics were identified in wild birds from different locations in Australia, with ducks feeding at a wastewater treatment plant harboring the greatest ARG burden^24^. Although several studies have identified antibiotic-resistant bacteria and ARGs in some lower vertebrates sampled from locations influenced by human activity^9,11,48^, far less is known about the resistome in wild lower vertebrates that live far from human habitation.

To the best of our knowledge, this study represents the first attempt to assess the functionally active resistome using meta-transcriptomics in a broad range of wildlife living from land to ocean, and that should rarely be subjected to direct human impact. However, they might be exposed to antibiotics and/or ARG contamination indirectly through contact with domestic animals and/or polluted environments. Even though we examined a relatively small number of samples per species, we revealed a remarkably high diversity and abundance of functional (i.e. expressed) ARGs in these lower vertebrates, comprising 194 ARGs that confer resistance to 13 antibiotic classes. This frequency of ARGs was comparable to that found in some environmental samples using the same database and metagenomic sequencing, such as drinking water (17 ARG types and 265 ARGs)^49^, pig fecal samples (18 ARG types and 257 ARGs)^50^, and samples from wastewater treatment plants (19 ARG types and 384 ARGs)^51^. Hence, these data imply that lower vertebrates are a major source and sink of ARGs in nature.

Also striking was that the diversity and composition of the vertebrate resistome varied with their ecological niche. A previous study noted differences in the prevalence of ARGs in wild mammals sampled from different habitats^47^. Similarly, ducks feeding at a wastewater treatment plant carried a higher level of ARG burden than birds sampled from other environments^24^. Notably, we observed that the most abundant resistome was in marine vertebrates, while the highest ARG diversity was present in land animals (Figs. 1 and 2). It may not be surprising to observe greater resistome diversity in terrestrial ecosystems as they are considered the major natural reservoirs of resistance genes due to the huge numbers of microbes they harbor^52^. In marked contrast it is striking that marine fish, even those living in the deep ocean (e.g. *Eptatretus burgeri* (average 116.6 RPKM) and *Chimaera* sp. (average: 194.4 RPKM)) possess highly abundant and functionally active ARG. Hundreds of various ARGs has been detected in various aquatic environments, including not only hospital and livestock wastewaters, but also surface water, groundwater and even drinking water^53^. Some ARGs encoding resistance to aminoglycoside^54^, chloramphenicol^55^ and tetracycline^56^ have also been found in marine waters or sediments. Despite the complicated spread pathways of ARGs in aquatic environments^53^, the majority of antibiotic-resistant bacteria harboring ARGs and residual antimicrobials from medicine and animal production will eventually flow into the sea^22,57^, thereby contaminating marine environments. Moreover, that some marine fish can move long distances makes it possible that they can disseminate ARGs globally in a similar manner to how birds and rodents spread microbial pathogens^58–60^. Combined, these data reinforce the need to better understand the circulation and evolution of antibiotic resistance in wildlife, especially marine animals.

Currently, ARGs are divided into two categories: intrinsic genes that evolved in nature to counteract the activity of natural antimicrobials produced by neighboring microbes (i.e., microbial interactions in nature), and genes that have been acquired by HGT since the use of antibiotics^29^. Generally, intrinsic genes show a specific association with their bacterial hosts, while such an association might be destructed by large amounts of acquired genes. As intrinsic genes are abundant in natural environments independently of antibiotic selection pressure^1,29^, it is perhaps unsurprising to observe significant correlations between the resistome and the active microbiome in terrestrial reptiles and amphibians. This is similar to results from other studies showing associations between the soil microbiome and resistome^44,61^, implying that most ARGs are intrinsic genes in terrestrial environments and their animal hosts, and natural evolution of ARGs are still dominated in these ecosystems. However, it is noteworthy that we revealed highly abundant and monotonous resistomes (i.e. with low ARG diversity) in marine fish that are ecologically distinct from animals present in other habitats. Such a separation was mainly driven by the extremely high proportion of a limited number of ARGs (e.g., *tet*A and several *bla*_TEM_ variants, Fig. S5) conferring tetracycline and beta-lactam resistance in marine fish. Notably, these ARGs were commonly associated with mobile elements (Table S5), suggesting that they were likely acquired through HGT. This result is also in accord with previous studies showing that tetracycline and beta-lactam resistance genes frequently transfer horizontally between bacteria in humans and animals, leading to the rapid spread of resistance phenotypes^27,44^. Moreover, our results also revealed a higher proportion of mobile ARGs in marine fish, and a loose correlation between the functionally resistome and the active microbiome in these animals as observed in aquatic environments^62^. These findings suggest a higher frequency of HGT of specific ARGs among animals in marine environments, which in turn led to similar resistome compositions in these animals. Hence, more attention should be paid to marine ecosystems as they may have a greater potential to disseminate ARGs through HGT.

Finally, these wild vertebrates also contained ARGs with high sequence similarity (99–100% amino acid identity) to clinical isolates of particular concern for public health. Among these, the *tet*A (YP_001687825), *bla*_TEM_ (e.g. GQ149347, DQ909059, AY040093) and *cat* (AF326777) genes are mobile ARGs and highly prevalent in the animals sampled, especially in marine fish. In addition, the mobile genes *aph* (3′′)-I (AF313472) encoding aminoglycoside resistance and *qnr*B (ABV66095) encoding quinolone resistance, as well as the intrinsic gene *bac*A (YP_002847905) encoding bacitracin resistance, were detected in reptile libraries. These results highlight the potential bidirectional exchange of resistance between wild animals and clinical isolates, similar to that between soil and clinical samples^63^. The dominance of *Enterobacteriaceae* in all habitats and the high number of mobile ARGs they carry suggests that these bacteria may be the main vectors for disseminating antibiotic resistance through HGT among the vertebrates sampled here^64–67^.

In conclusion, these data reveal the remarkable diversity and transcriptional levels of ARGs in a broad range of wild lower vertebrates from ocean to land, and suggest that these wildlife species might play an important role in the global spread of ARGs. However, because of the relatively small and uneven sample size from different habitats and only a subset of animal species as representatives for each of habitats, it is clear that additional work is needed to confirm the abundance and dissemination of ARGs in a broader range of wildlife species. In sum, this study highlights that more attention should be paid to understanding the extent and pattern of antimicrobial resistance in wildlife, particularly in marine ecosystems.

## Methods

### Meta-transcriptomic data collection and analysis

We aimed to investigate the antibiotic resistome in vertebrate species that have rarely been analyzed for resistance genes. Accordingly, we focused on fish, amphibians and reptiles rather than mammals and birds that have been wildly studied. Critically, the use of meta-transcriptomics using bulk RNA sequencing allows us to characterize the entire set of functionally active genes. A total of 46 available vertebrate meta-transcriptomic libraries were selected from our previous study^42^ based on the following criteria: (a) samples have clear information on taxon, sampling date and location, (b) each sampling site contains more than one vertebrate class (with the exception of Chile and Nigeria—see Supplementary Methods), and (c) each of the libraries includes no more than two vertebrate species. The selected libraries comprise over 25 vertebrate species from seven vertebrate classes in the phylum Chordata (Table S1). These libraries comprised wild lower vertebrates from land to the deep ocean, collecting from Guangdong, Guangxi and Hubei provinces in the Chinese mainland, the Eastern China Sea and South China Sea of China, as well as from Chile and Nigeria.

As described previously^42^, RNA was extracted from individual animal tissues (gut, liver, gill and lung), followed by library construction using pooled RNA extractions (Table S1). All the libraries were subject to 150 bp paired-end sequencing on the HiSeq 4000 platform (Illumina), generating a total of 184 million sequence reads^42^. For each RNA library, the sequence reads with adaptor- and quality-filtered were assembled de novo using the Trinity program (version 2.5.1)^68,69^. The contigs generated were used for screening of ARG contigs and assessment of their mobility (see below). More detailed information about data collection and processing can be found in Supplementary Methods.

### Identification and characterization of ARGs

ARGs were firstly identified by using Diamond Blastx^43^ against the expanded Structured Antibiotic Resistance Genes database (SARG version 2.0)^70^ with an *e*-value cutoff set at 1 × 10^–7^. A relatively strict criteria for ARG read annotation with a sequence identity of 90% and an alignment length of more than 25 amino acids was used to reduce the number of false-positives^71–73^. To compare gene expression across libraries, ARG abundance was calculated as RPKM to account for gene length and library size^44^, and was further normalized by a stably expressed host gene^24,74^ (Supplementary Methods and Fig. S1).

Although it is possible that there are laboratory contaminants in these data, we retained genes (i.e., *tet*A, *bla*_TEM-1_, *bla*_TEM-157_, *bla*_TEM-171_ and *bla*_TEM-197_) that were found in all the sequencing libraries since they exhibited markedly different patterns of abundance between libraries (Table S2). In future studies a negative and/or a positive control sequencing library should be used to help exclude possible laboratory contamination.

### Alpha and beta diversity

To calculate alpha diversity, the raw ARG count matrix was rarefied based on the lowest samples’ depth using the *rarefy* function in the *vegan* package in R (version 3.5.1)^75^. Species richness and diversity indexes (Shannon and Simpson index) were then calculated based on the rarefied count matrix using the *diversity* function in vegan^76^. Importantly, the diversity of ARGs in individual vertebrate species was validated using PCR targeting 20 resistance genes (see Supplementary Methods; Table S8).

For beta diversity, a Bray–Curtis dissimilarity matrix was calculated based on the relative abundance of ARGs and was subject to Nonmetric Multidimensional Scaling (NMDS) to examine the clustering patterns of ARGs. This was done using the *metaMDS* function in the *vegan* package.

### Characterization of active bacterial communities

We aimed to investigate how the active microbiome and their association with the functional resistome varied across habitats. We therefore assessed the structure and clustering patterns of the active bacterial community based on relative abundance, rather than calculating their absolute abundance. To characterize active bacterial communities, a local 16S rRNA database was constructed by extracting all the 16S rRNA-related sequences from the SILVA database^77^. One million reads from each meta-transcriptomic library were randomly subsampled and searched (using blastn) against the local 16S rRNA database to exact all reads corresponding to 16S rRNA genes (70% identity and *e*-value 10^–10^). A taxonomic classification of these exacted 16S rRNA reads was then performed using the RDP classifier (version 2.10.1). Relative abundance of bacterial genera was calculated as genus read counts divided by the total bacterial read counts in the library. Since these data depict transcriptionally active 16S rRNA, not the underlying 16S rRNA genes, they do not necessarily represent the entire set of the bacterial communities.

### Taxonomic annotation of ARG contigs

The bacterial hosts of ARGs were predicted using the following strategy. First, the assembled contigs were aligned to the SARG database using Diamond Blastx with an *e*-value of 10^–10^ and a sequence identity above 80%^62,78^. To minimize false positives, a query contig was only considered in the downstream analysis if it overlapped the subject resistance gene with at least 20% coverage^24^. Open reading frames (ORFs) in the resultant ARG contigs were predicted using ORFfinder (version 0.4.3), and were then compared (*e*-value 10^–7^) against the entire NCBI-NR database to further eliminate false-positive ARG contigs and to predict their phylogenetic origin. Taxonomic annotation was performed based on the NCBI taxonomy using the *getTaxonomy* function in the *taxonomizr* package in R. An ARG contig was assigned to a bacterial taxon if the largest number of its ORFs (excluding those that are unclassified) were classified into the same bacterial kingdom/phylum/class/order/family/genus^62^.

### Evaluation of ARG mobility

Potential ARG mobility was evaluated as reported previously^41,44^. All the ORFs in ARG contigs were annotated by searching against the NCBI-NR, PFAM^79^ and the TIGRFAMS databases^80^ with an *e*-value of 10^–7^. Putative MGEs were identified according to the ORF annotation based on string matches to one of the following keywords: “transposase”, “transposon”, “integrase”, “integron”, “conjugative”, “conjugal”, “recombinase”, “recombination”, “mobilization”, “phage”^61^. A resistance gene was considered co-localized with a MGE if an ARG contig was shared with a MGE ORF. In addition, all of the ARG contigs identified were aligned (using blastn) to plasmid genomes downloaded from the NCBI Refseq database with a sequence identity above 95% and a subject coverage of at least 20% (*e*-value 10^–10^)^41^.

### Statistical analysis

Differences in ARG abundance, diversity and the number of genes successfully amplified by PCR between groups were accessed statistically using a nonparametric Kruskal–Wallis test in R. Permutational multivariate analysis of variance based on the Bray–Curtis dissimilarity matrix of ARGs and bacterial genus was performed using the *adonis2* function in R. A Mantel test was performed using two Bray–Curtis dissimilarity matrices based on both Pearson and Spearman’s rank correlation with the *vegan* package in R, with *p* values determined from 9999 permutations. A Procrustes analysis was conducted in QIIME (using the script transform_coordinate_matrices.py), with two Bray–Curtis distance plots built from ARG and bacterial relative abundance, with M^2^ and *p*-value determined from 9999 permutations^61,81^. The R packages *ggplot2* and *pheatmap* were used to produce the NMDS plots, heatmaps, scatter dot plots and stacked columns.

### Ethics approval

The procedures for sampling and sample processing were approved by the ethics committee of the National Institute for Communicable Disease Control and Prevention of the Chinese CDC. All experiments were performed in accordance with relevant guidelines and regulations.

## Supplementary information

Supplementary Information 1.

Supplementary Information 2.

## Data Availability

Raw sequence reads used in this study are available at the NCBI Short Read Archive (SRA) database under the BioProject accession number PRJNA418053 (Table S1)^42^.
