# Supplementary material for: Diverse and abundant resistome in terrestrial and aquatic vertebrates revealed by transcriptional analysis
Source: Sci Rep. 2020 Nov 2;10:18870. doi: 10.1038/s41598-020-75904-x (PMC7608656; doi:10.1038/s41598-020-75904-x)
Supplement: Supplementary file 2 — Supplementary Information 2. [file 41598_2020_75904_MOESM2_ESM.docx]

**Supplementary Materials For**

**Diverse and abundant resistome in terrestrial and aquatic vertebrates revealed by transcriptional analysis**

Yan-Mei Chen^1,2^, Edward C. Holmes^2,4^, Xiao Chen^5^, Jun-Hua Tian^6^, Xian-Dan Lin^7^, Xin-Cheng Qin^3^, Wen-Hua Gao^3^, Jing Liu^3^, Zhong-Dao Wu^1^#, Yong-Zhen Zhang^2^#

^1^Zhongshan School of Medicine, Sun Yat-sen University, Guangzhou, 510080, China.

^2^Shanghai Public Health Clinical Center, Fudan University, Shanghai, China.

^3^Department of Zoonosis, National Institute for Communicable Disease Control and Prevention, Chinese Center for Disease Control and Prevention, Changping, Beijing, China.

^4^Marie Bashir Institute for Infectious Diseases and Biosecurity, School of Life and Environmental Sciences and School of Medical Sciences, The University of Sydney, Sydney, Australia.

^5^College of Marine Sciences, South China Agricultural University, Guangzhou, Guangdong, China.

^6^Wuhan Center for Disease Control and Prevention, Wuhan, Hubei, China

^7^Wenzhou Center for Disease Control and Prevention, Wenzhou, Zhejiang, China.

Corresponding author: Dr. Yong-Zhen Zhang, Email: zhangyongzhen@shphc.org.cn; or Zhong-Dao Wu, Email: [wuzhd@mail.sysu.edu.cn](mailto:wuzhd@mail.sysu.edu.cn)

**Supplementary Methods**

**Sample selection**

A total of 46 vertebrate meta-transcriptomic libraries were selected from our previous study^1^ based on the criteria listed in the main text. Although only the vertebrate class Sarcopterygii (i.e. lungfish) were collected in Chile and Nigeria, we retained these data as they were the only samples obtained outside of China (Table S1). All of the samples were kept at -20°C during transit and were transferred to -80°C for storage.

As we reported previously^1^, internal organs including the gut, liver and gill were harvested for cartilaginous and jawless fish, while gut, liver and lung samples were obtained for amphibians and reptiles. For lungfish, all four tissue types (gut, liver, lung and gill) were harvested. For lancelets, the entire individual was used due to their small body size. For ray-finned fish, only two gill libraries meet the screening criteria and were selected. All animal specimens were stored at -80°C before RNA extraction.

**RNA library preparation and sequencing**

Total RNA was extracted from homogenates of individual animal specimens as previously reported^1^. Aliquots of RNA solutions from several individuals of a particular taxonomic group were then pooled in equal quantity and libraries constructed using the TruSeq total RNA library preparation protocol (Illumina). Host rRNA, including cytoplasmic rRNA and mitochondrial rRNA, was removed using the Ribo-Zero-Gold kit (Human/Mouse/Rat, Illumina). Paired-end (150bp) sequencing was performed on a HiSeq4000 platform. The size of each library is provided in Table S1.

**Data processing**

For each library, sequencing reads were adaptor- and quality-trimmed with Trimmomatic followed by *de novo* assembly using the Trinity program (version 2.5.1) with default parameter settings. The Trinity program is an efficient and robust method for the *de novo* assembly of transcriptomes from RNA-seq data^2,3^ and has been commonly used in previous studies^1,4,5^. No filtering of host or bacterial reads was performed prior to assembly.

**Estimation of ARG abundance**

We used the expanded Structured Antibiotic Resistance Genes database (SARG version 2.0)^6^ for ARG annotation as this is a comprehensive resistance genes database integrating the two most commonly used databases - the Antibiotic Resistance Genes Database (ARDB)^7^ and the Comprehensive Antibiotic Resistance Database (CARD)^8,9^ - as well as sequences retrieved and validated from the non-redundant protein database (NCBI-NR).

Gene expression normalization was performed using the RPKM (reads per kilobase per million reads) method^10^, combined with the use of stably expressed host genes^11,12^. We used a host rather than a bacterial gene because our aim was to assess the ARG abundance ‘per host’ rather than ‘per bacteria cell’. Gene abundances were calculated as (1):

$$\frac{{RPKM}_{ARG}}{{RPKM}_{host}}$$

in which *RPKM_ARG_* is the ARG expression and *RPKM_host_* is the expression of a stably expressed host gene.

RPKM is calculated as (2):

$$\frac{N_{reads}}{gene length \times library size}\times{10}^{9}$$

in which *N_reads_* is the number of mapped reads to the gene, *gene length* is the length of the gene in base-pairs, and *library size* is the total number of reads in the corresponding library.

Five host reference genes, including the ribosomal protein S13 (RPS13), ribosomal protein S23 (RPS23), ribosomal protein L32 (RPL32), Elongation factor 1 alpha complex (EF1a), and NADH dehydrogenase 1 alpha subcomplex (NDUFA) were selected as previous studies have shown that they are stably expressed^12-14^. Reads were mapped to assembled contigs annotated to these genes using bowtie2^15^, and the number of mapped reads was obtained by samtools^16^. We also attempted to find the reference sequences of these genes for read mapping. However, as no reference genome or gene sequence were available for several vertebrate orders (even for classes), we used the assembled contigs for host gene abundance estimation. The average number of mapped reads and average length of contigs annotated to the gene was used as *N_reads_* and *gene length* as provided in equation (2). Notably, not all these genes were stably expressed in all libraries (Fig. S1). Therefore, only the best one (NDUFA) was used in subsequent analysis.

**Evaluating ARG richness in individual animal specimens by PCR**

Each RNA library comprised several individuals of the same taxonomic group sampled from the same site. PCR assays were performed on individual animal specimens to confirm the results obtained from the meta-transcriptomic data. Accordingly, 20 RNA libraries were selected from different tissue types in different animal classes, with the exception of jawless fish and lancelets for which no RNA solutions remained in our laboratory (Table S3). We targeted 22 ARGs that were (i) found in at least one of the seven animal classes in the meta-transcriptomic libraries, and (ii) presented as many ARG types as possible. We designed primers based on both the reference sequences in the database and the contigs of each gene. Considering that the RNA solutions were extracted approximately two years ago, we also amplified two viral fragments that were found in two libraries from our previous study^1^ to assess the RNA quality and consider as positive controls (Table S8). Total RNA was first reverse transcribed using one step RT-PCR kit (TaKaRa), followed by nested PCR targeting the conserved regions of each ARG or virus. The target PCR products were then assessed in 2% agarose gels and further validated by Sanger sequencing. A total of 21 ARGs were successfully amplified. However, because the *bla*_TEM_ and *cat* genes were amplified in all RNA solutions and in negative controls they were excluded from the PCR assays.

**References**

1 Shi, M. *et al.* The evolutionary history of vertebrate RNA viruses. *Nature* **556**, 197-202 (2018).

2 Grabherr, M. G. *et al.* Full-length transcriptome assembly from RNA-Seq data without a reference genome. *Nat Biotechnol* **29**, 644-652 (2011).

3 Haas, B. J. *et al.* *De novo* transcript sequence reconstruction from RNA-seq using the Trinity platform for reference generation and analysis. *Nat Protoc* **8**, 1494-1512 (2013).

4 Shi, M. *et al.* Redefining the invertebrate RNA virosphere. *Nature* **540**, 539-543 (2016).

5 Babb, P. L. *et al.* The Nephila clavipes genome highlights the diversity of spider silk genes and their complex expression. *Nat Genet* **49**, 895-903 (2017).

6 Yin, X. *et al.* ARGs-OAP v2.0 with an expanded SARG database and hidden markov models for enhancement characterization and quantification of antibiotic resistance genes in environmental metagenomes. *Bioinformatics* **34**, 2263-2270 (2018).

7 Liu, B. & Pop, M. ARDB—Antibiotic resistance genes database. *Nucleic Acids Res* **37**, D443-447 (2009).

8 McArthur, A. G. *et al.* The comprehensive antibiotic resistance database. *Antimicrob Agents Chemother* **57**, 3348-3357 (2013).

9 Jia, B. *et al.* CARD 2017: expansion and model-centric curation of the comprehensive antibiotic resistance database. *Nucleic Acids Res* **45**, D566-573 (2017).

10 Mortazavi, A., Williams, B. A., McCue, K., Schaeffer, L. & Wold, B. Mapping and quantifying mammalian transcriptomes by RNA-Seq. *Nat Methods* **5**, 621 (2008).

11 Evans, C., Hardin, J. & Stoebel, D. M. Selecting between-sample RNA-Seq normalization methods from the perspective of their assumptions. *Brief Bioinform* **19**, 776-792 (2017).

12 Marcelino, V. R. *et al.* Meta-transcriptomics reveals a diverse antibiotic resistance gene pool in avian microbiomes. *BMC Biol* **17**, 31 (2019).

13 Chapman, J. R. *et al.* A panel of stably expressed reference genes for real-time qPCR gene expression studies of mallards (Anas platyrhynchos). *PLos One* **11**, e0149454 (2016).

14 Fu, W. *et al.* Exploring valid reference genes for quantitative real-time PCR analysis in *Plutella xylostella* (Lepidoptera: Plutellidae). *Int J Biol Sci* **9**, 792-802 (2013).

15 Langmead, B. & Salzberg, S. L. Fast gapped-read alignment with Bowtie 2. *Nat Methods* **9**, 357 (2012).

16 Li, H. *et al.* The sequence alignment/map format and SAMtools. *Bioinformatics* **25**, 2078-2079 (2009).

**Supplementary Figures**

**
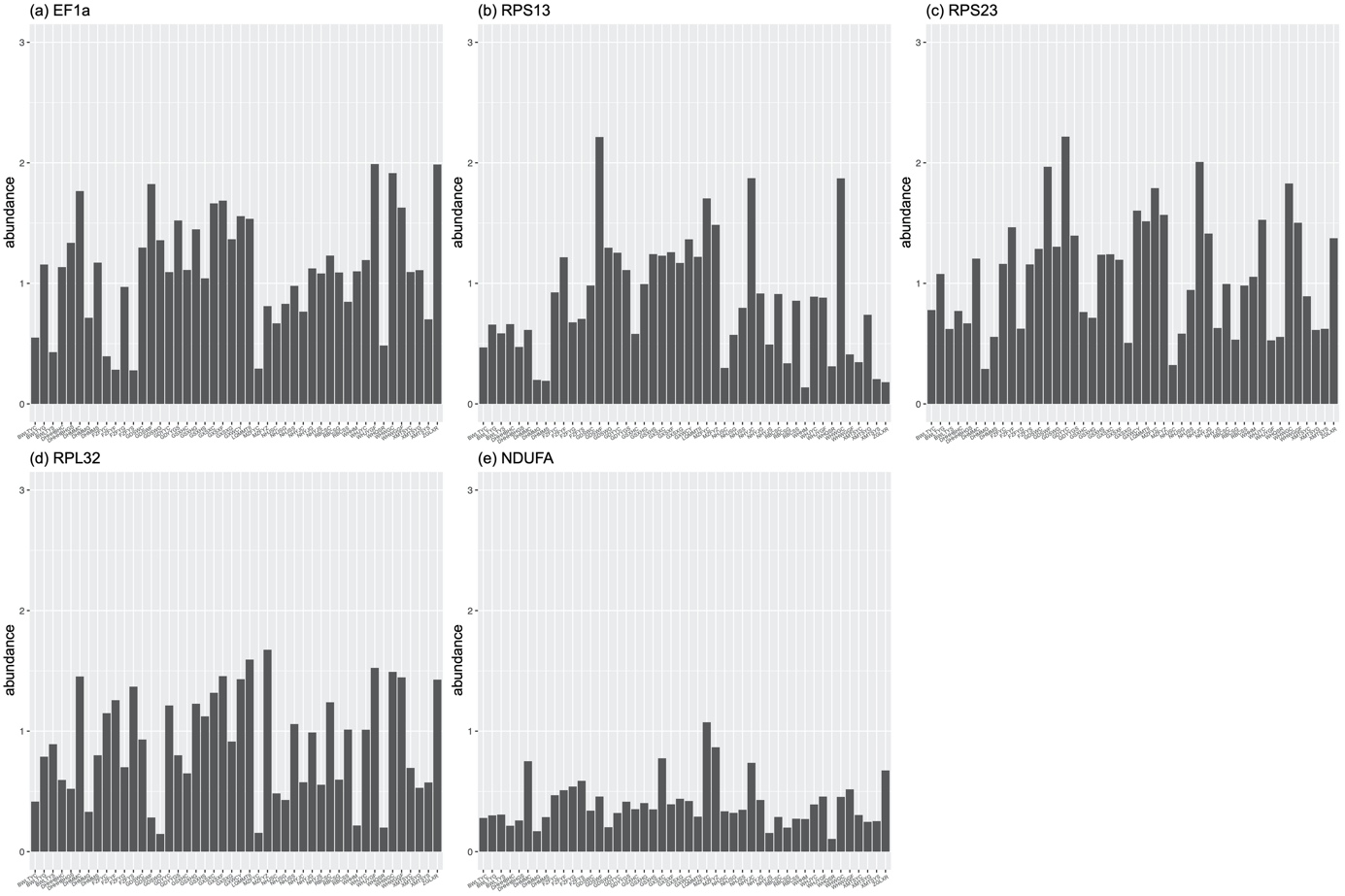
**

**Figure S1.** Abundance of five host reference genes. Abundance were calculated as RPKM and were log transformed in the bar plot. These genes were previously shown to be stably expressed in other studies and hence were utilized here. However, not all these genes were stably expressed in all libraries and therefore only the best, NDUFA, was considered in the downstream analysis.

**
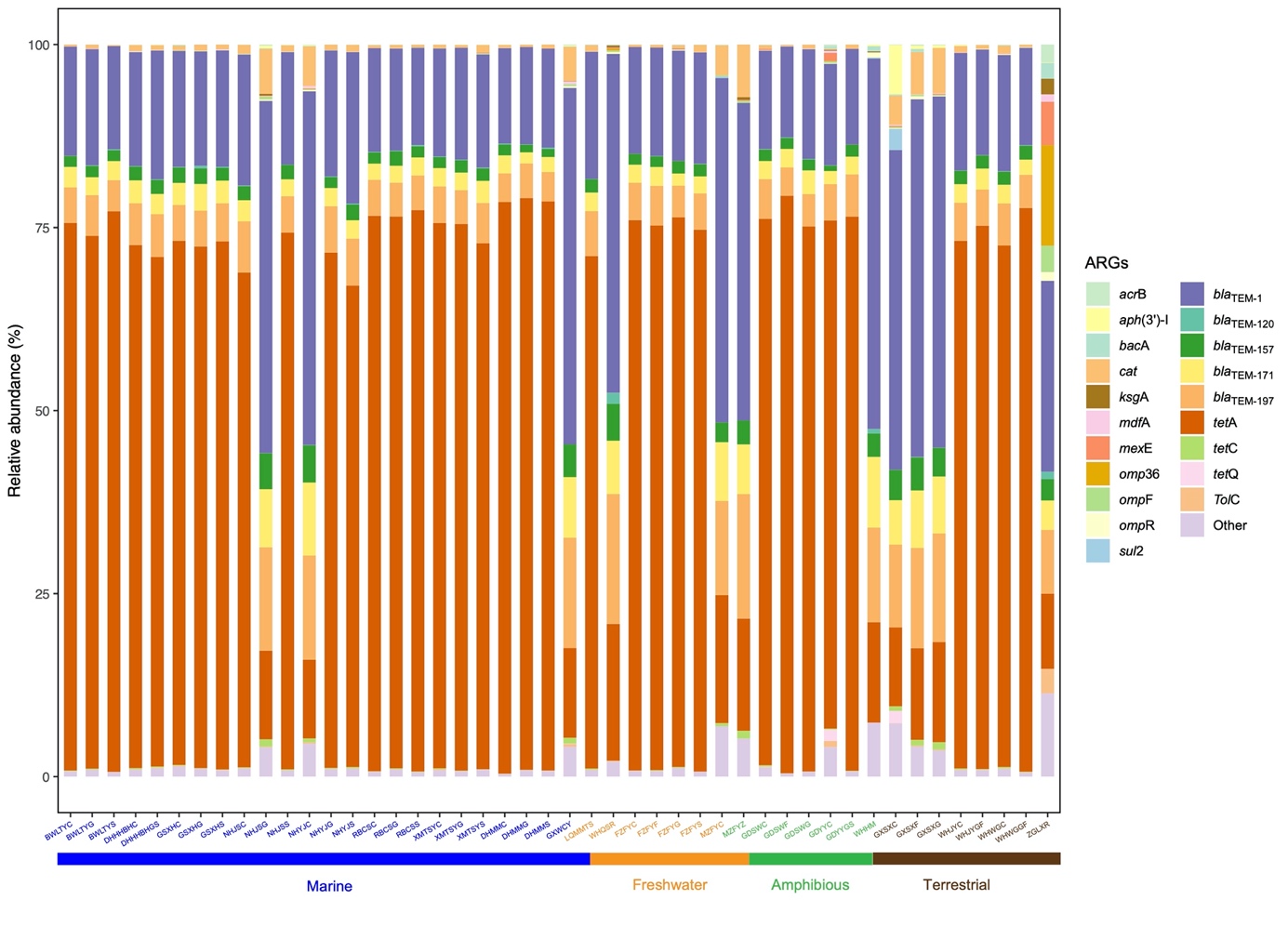
**

**Figure­ S2.** Relative abundance of the 20 most common ARGs in each library.

**
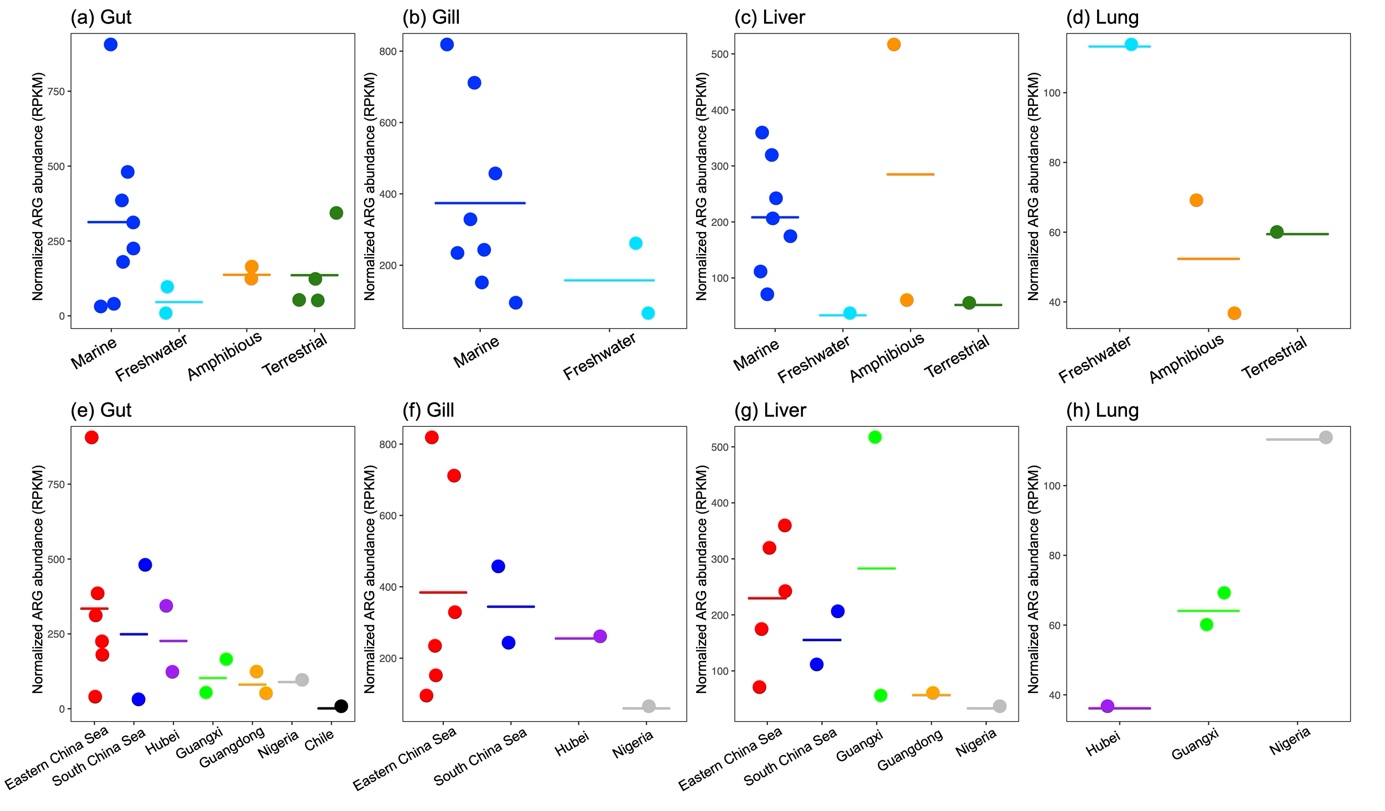
**

**Figure S3.** Total ARG abundance of each habitat **(a-d)** and sampling site **(e-h)** in individual tissue types. Each dot represents a meta-transcriptomic library. Cross bars indicate the mean values. No statistical analysis was performed due to the small number of libraries per tissue type per habitat/site.


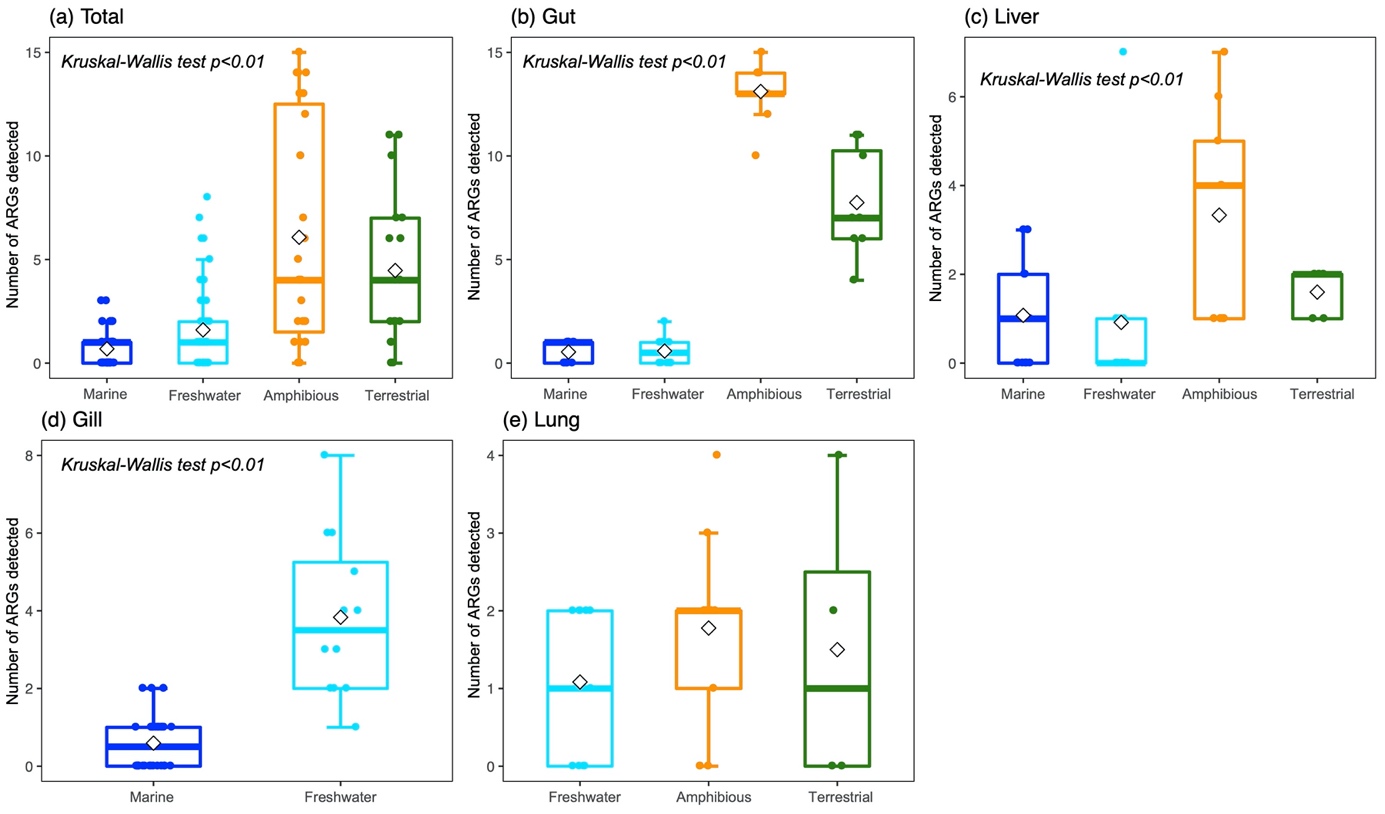
**Figure S4**. PCR assays using individual animal specimens confirmed that terrestrial and amphibious vertebrates have greater ARG diversity, while a lower diversity is observed in marine fish. Differences between habitats were assessed in total specimens **(a)** and each tissue type, including gut **(b)**, liver **(c)**, gill **(d)** and lung **(e)** using a Kruskal-Wallis test. Each dot represents an individual specimen. The horizontal box lines represent the first quartile, the median, and the third quartile. Whiskers denote the range of points within the first quartile − 1.5× the interquartile range and the third quartile + 1.5× the interquartile range. Diamonds represent the mean values.

**
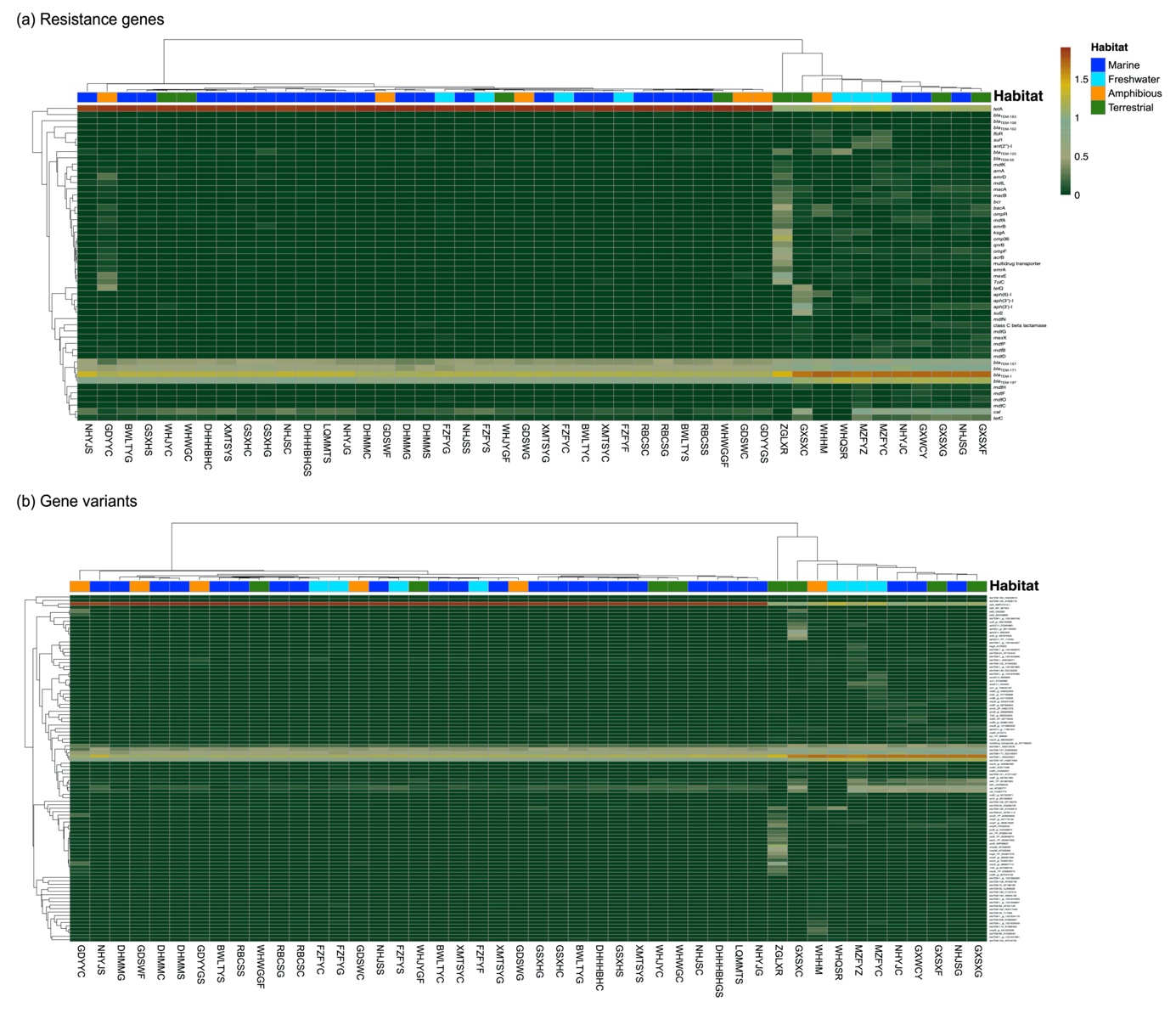
**

**Figure S5**. ­­­­­Heat map of resistance gene **(a)** and gene variant **(b)** levels. Colors represent log transformed relative abundances. Bray-Curtis dissimilarity and Pearson correlation coefficients were used to hierarchically cluster (using the UPGMA method) samples and ARGs, respectively.

**
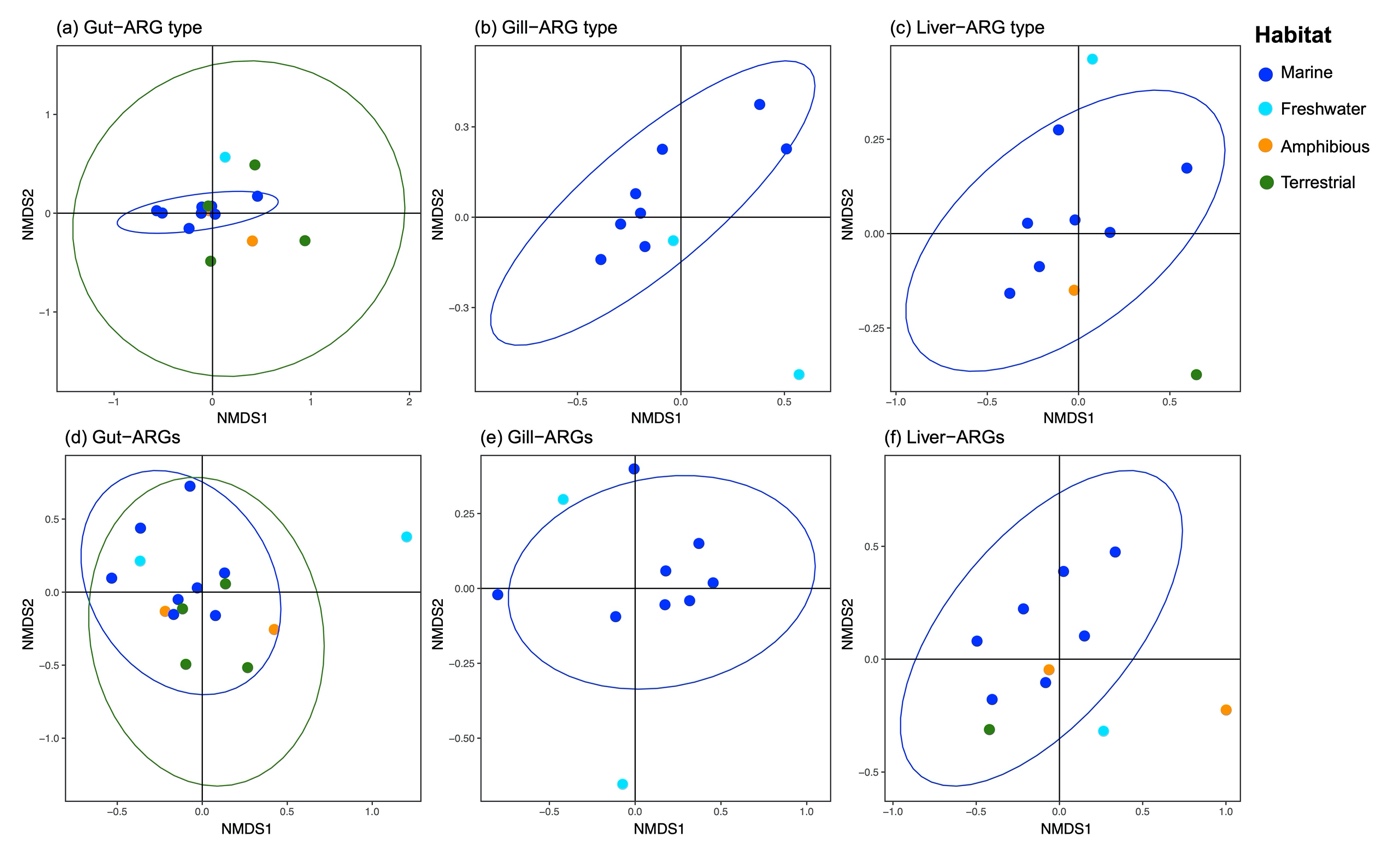
**

**Figure S6**. Non-metric multidimensional scaling (NMDS) analysis based on both ARG type **(a-c)** and gene levels **(d-f)** in individual tissues revealed the clear separation of the marine fish resistome. Each dot represents a meta-transcriptomic library. Ellipses were drawn at a confidence level of 0.95. No statistical analysis was performed due to the small number of samples in some groups.


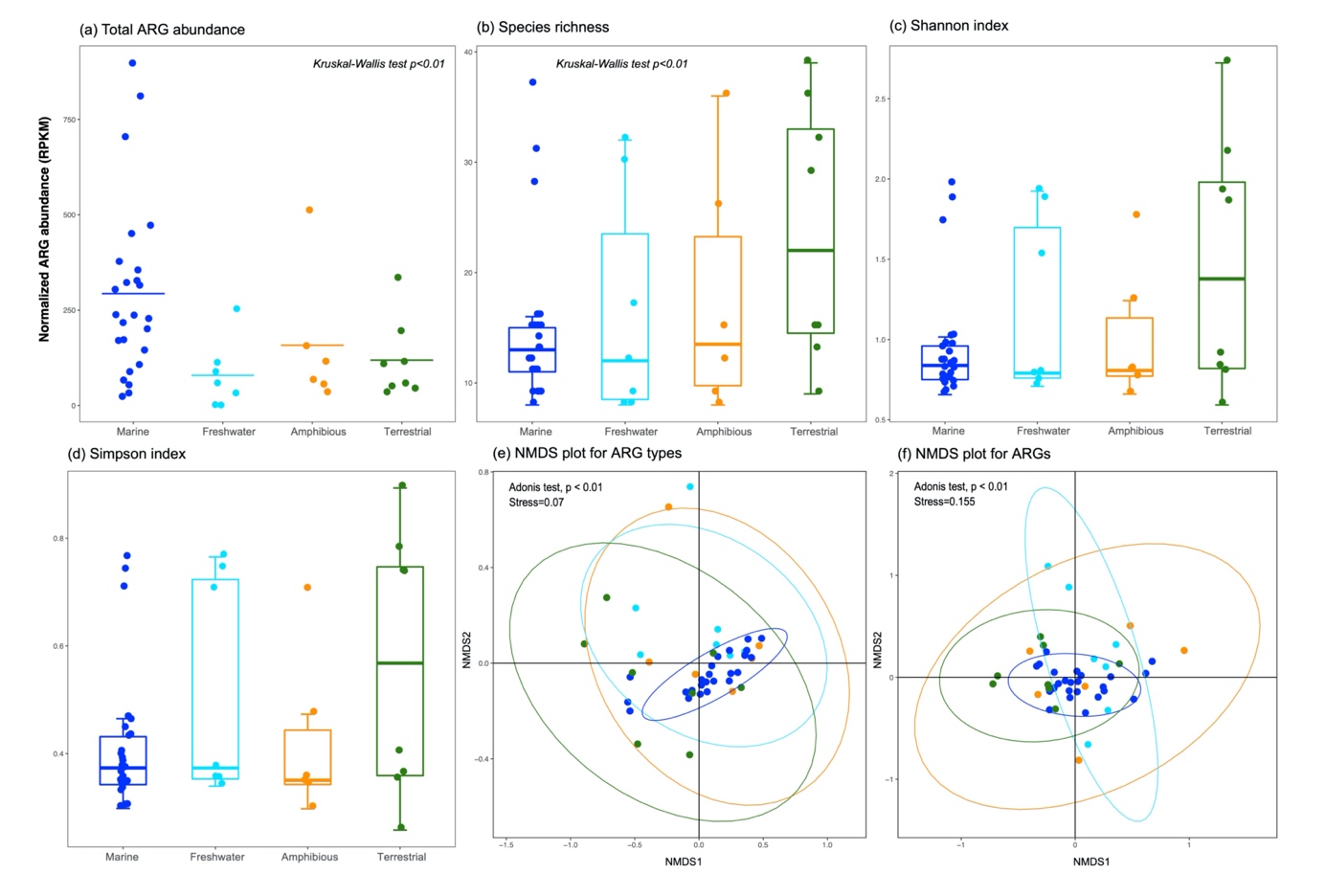


**Figure S7**. ARG abundance, diversity and clustering patterns across habitats. The *omp*36 and *omp*F genes that can cause resistance only when there are mutated alleles present were excluded. Each dot represents a meta-transcriptomic library. Cross bars in the dot plot indicate the mean values. In the box plot, the horizontal box lines represent the first quartile, the median, and the third quartile; whiskers denote the range of points within the first quartile − 1.5× the interquartile range and the third quartile + 1.5× the interquartile range. In the NMDS plot, ellipses were drawn at a confidence level of 0.95.
